# The Current Role of Imaging in the Diagnosis of Inflammatory Bowel Disease and Detection of Its Complications: A Systematic Review

**DOI:** 10.7759/cureus.73134

**Published:** 2024-11-06

**Authors:** Muhammad Yasir Younis, Muhammad Usman Khan, Usman Khan, Talal Latif Khan, Hassan Mukarram, Kanav Jain, Insha Ilyas, Wachi Jain

**Affiliations:** 1 Gastroenterology, Lahore General Hospital, Lahore, PAK; 2 Gastroenterology, Allama Iqbal Teaching Hospital Dera Ghazi Khan, Dera Ghazi Khan, PAK; 3 Medicine, Sir Ganga Ram Hospital, Lahore, PAK; 4 Medicine, North Devon District Hospital, Barnstaple, GBR; 5 Gastroenterology, Services Institute of Medical Sciences, Lahore, PAK; 6 Medicine, Countess of Chester Trust, Chester, GBR; 7 Medicine, Lincoln County Hospital, Lincoln, GBR

**Keywords:** complications, crohn's disease, diagnosis, imaging modalities, inflammatory bowel disease, ulcerative colitis

## Abstract

Inflammatory bowel disease (IBD) encompasses complex gastrointestinal (GI) conditions, primarily Crohn's disease (CD) and ulcerative colitis (UC), requiring precise imaging for effective diagnosis and management of complications. This systematic review aimed to evaluate the current role of imaging modalities in diagnosing IBD and detecting related complications. The review adhered to the Preferred Reporting Items for Systematic Reviews and Meta-Analyses (PRISMA) guidelines. We performed a literature search using text words and controlled vocabulary applying Boolean operators "AND," "OR," with various combinations on databases such as PubMed, Embase, and Cochrane Library. The search targeted open-access articles involving humans, with full-text available, and published in the English Language from 2005 to 2024. The quality of the included studies was assessed using the Cochrane Risk-of-Bias (RoB) checklist.

Our search process identified 127 records from Cochrane (39), Embase (29), and PubMed (59). After removing 98 irrelevant records, 29 underwent further screening. Five were excluded as they involved irrelevant problems or outcomes, leaving us with 24 reports with full text, all of which were accessible. Following the eligibility assessment, two more reports were excluded due to inaccessibility, and 22 studies were included in the final analysis. The risk of bias and methodological quality assessment revealed that out of 22 studies analyzed, five (23%) had a high risk of bias, while 13 (59%) were classified as moderate risk, and four (18%) showed low risk. This distribution highlights a predominance of moderate-risk studies in research on imaging in IBD, emphasizing the need for enhanced study designs in future investigations.

Our findings revealed the varying effectiveness of imaging modalities in diagnosing complications of CD and UC. Magnetic resonance enterography (MRE) stands out as the preferred method for CD due to its high sensitivity and noninvasive nature. In contrast, colonoscopy remains the gold standard for UC, providing direct visualization of mucosal lesions. While techniques like ultrasound and capsule endoscopy offer valuable insights, they have limitations that may affect their utility in certain cases.

## Introduction and background

Inflammatory bowel disease (IBD) refers to chronic inflammatory conditions affecting the gastrointestinal (GI) tract [1]. It is classified into two main types: Crohn's disease (CD) and ulcerative colitis (UC). While both conditions cause prolonged inflammation of the GI tract, they differ in terms of the areas affected, the depth of inflammation, and clinical presentation [2]. CD is a chronic inflammatory disorder that affects any part of the GI tract, from the mouth to the anus. However, it most commonly involves the terminal ileum and the colon. The inflammation is typically transmural, affecting all bowel wall layers and causing complications such as strictures, fistulas, and abscesses [3]. CD is more common in Western countries, particularly in North America and Northern Europe, with a yearly incidence rate of 3-20 per 100,000 people [4].

UC is a chronic inflammatory condition that primarily affects the colon and rectum, with inflammation confined to the mucosal layer of the bowel wall. It is more prevalent in Western countries, with annual incidence rates ranging from 6 to 15 per 100,000 people [5]. CD manifests as abdominal pain, diarrhea, weight loss, malnutrition, fever and fatigue, perianal disease, and extra-intestinal manifestations such as arthritis, uveitis, erythema nodosum, and primary sclerosing cholangitis (PSC). Common complications include strictures, fistulas, and abscesses [6,7]. UC is associated with symptoms such as bloody diarrhea, rectal pain and urgency, abdominal pain, fatigue and fever, and extra-intestinal manifestations affecting the skin, eyes, liver (especially PSC), and joints. Its complications include toxic megacolon, colorectal cancer, perforation, and severe bleeding [8,9]. CD and UC are both GI diseases with similar symptoms, such as diarrhea, rectal bleeding, and abdominal pain [10].

Some patients may present with both conditions, making diagnosis difficult. The challenges associated with imaging in these patients include endoscopic limitations, non-specific findings, and delays in diagnosis due to non-classic presentations and misdiagnosis. Imaging modalities like magnetic resonance enterography (MRE), CT enterography (CTE), ultrasound (US), and endoscopy are essential for the diagnosis and monitoring of complications [11,12]. Early and accurate detection of complications like strictures, fistulas, abscesses, and colorectal cancer is critical for managing IBD, guiding treatment decisions, and improving patient outcomes [13]. Accurate diagnosis and monitoring of disease progression are key in IBD. Imaging modalities have been developed to aid in the diagnosis, staging, and detection of complications associated with IBD. However, there is no consensus on the optimal use and timing of these imaging tools, particularly in terms of disease monitoring, treatment response, and predicting outcomes [14].

There are several gaps in the literature related to the above-mentioned aspects pertaining to imaging, including a lack of comparative studies, inconsistent guidelines for imaging use, variability in imaging accessibility, and limited focus on emerging technologies. In light of this, we conducted this systematic review of the role of imaging in diagnosing and monitoring IBD to address these study gaps and provide evidence-based recommendations for clinical practice. By synthesizing data on the performance, benefits, and limitations of various imaging modalities, we believe that such a review can help standardize protocols, improve patient outcomes, and identify areas where further research is needed.

## Review

Methodology

We conducted a systematic review using the Preferred Reporting Items for Systematic Reviews and Meta-Analyses (PRISMA) guidelines [15]. The research question was formulated using the PICO (Population, Intervention, Comparison, and Outcome) framework [16].

PICO Framework

Table 1 outlines the PICO framework employed to structure the review of studies on the current role of imaging in the diagnosis of and detection of complications in IBD.

**Table 1:** PICO framework CTE: computed tomography enterography; MRE: magnetic resonance enterography; PICO: Population, Intervention, Comparison, and Outcome

Concepts	Text words	Controlled vocabulary
Population: patients diagnosed with inflammatory bowel disease (IBD)	"Inflammatory Bowel Disease," "IBD," "Crohn's disease," "Ulcerative colitis"	"Inflammatory Bowel Disease", "IBD" [Mesh]
Intervention: utilization of imaging modalities such as MRE, CTE, ultrasound, capsule endoscopy, and diffusion-weighted MRI for diagnosis and monitoring	"Magnetic resonance enterography (MRE)," "Computed tomography enterography (CTE)," "Ultrasound," "Capsule endoscopy," "Diffusion-weighted MRI," "IBD imaging"	"Imaging Techniques", "Magnetic Resonance Imaging" [Mesh], "Computed Tomography" [Mesh]
Comparison: comparison of traditional methods like endoscopy	"Traditional methods," "Endoscopy," "Colonoscopy," "Alternative management"	“Endoscopy”, “Traditional Management” [Mesh], “Alternative Management Approaches”
Outcomes: improved diagnostic accuracy, detection of complications	"Improved diagnostic accuracy," "Detection of complications"	“Complications”, “Management Strategies”, “Diagnostic Accuracy” [Mesh]

Research Question

“How do advanced imaging modalities, such as MRE, CTE, ultrasound, and diffusion-weighted MRI, compare to traditional endoscopic methods in diagnosing and detecting complications in patients with inflammatory bowel disease?"

Search Strategy and Search Terms

A detailed search strategy was employed to explore the field of lung transplantation, focusing on complications and current management. The literature search focused on studies involving various imaging modalities, such as MRE, CTE, ultrasound, capsule endoscopy, and diffusion-weighted MRI. Boolean operators (AND, OR) were used to combine these terms, and the search was conducted on databases such as PubMed, Embase, and the Cochrane Library.

Search String

(“Inflammatory Bowel Disease” OR “IBD” OR “Crohn’s Disease” OR “Ulcerative Colitis”)

AND (“Magnetic Resonance Enterography” OR “MRE” OR “Computed Tomography Enterography” OR “CTE” OR “Ultrasound” OR “Capsule Endoscopy” OR “Diffusion-Weighted MRI” OR “Imaging Techniques”) AND (“Endoscopy” OR “Colonoscopy” OR “Traditional Methods”) AND (“Diagnostic Accuracy” OR “Complication Detection” OR “Disease Monitoring” OR “Bowel Strictures” OR “Fistulas” OR “Abscesses”)

Inclusion Criteria

This review focused on recent studies involving individuals diagnosed with IBD, including CD and UC, to assess the current role of imaging modalities in the diagnosis and detection of complications. Studies evaluating the effectiveness of imaging techniques such as MRE, CTE, ultrasound, capsule endoscopy, and diffusion-weighted MRI were included. Studies comparing these imaging techniques with traditional diagnostic approaches, like endoscopy, were also considered. Key outcomes of interest were diagnostic accuracy, early detection of complications (e.g., strictures, fistulas, and abscesses), and monitoring disease progression.

To ensure relevance and quality, only studies published in the last 15 years, written in English, and involving human subjects were included. Open-access, full-text articles were prioritized to ensure accessibility. This inclusion criterion ensured that the most current, high-quality, and relevant research was included, providing a comprehensive review of the evolving role of imaging in IBD management.

Exclusion Criteria

Cohort, case-control, and observational studies, as well as case reports and case series, were excluded due to concerns about their limited generalizability. Conference abstracts were not considered, as they typically lack detailed methodologies and complete results. Editorials, letters, and review articles, including systematic reviews and meta-analyses, were excluded since they do not provide original research data. To maintain a focus on the adult population, studies involving teenagers, children, and animals were not included. Articles published before 2005 were excluded to ensure the inclusion of recent and up-to-date research practices. Additionally, studies with restricted data access, incomplete analysis, or those requiring paid access were omitted to avoid biases related to accessibility and to promote inclusivity. This strategy ensured that only high-quality, relevant, and recent research on IBD imaging techniques was considered for the review.

Study Selection Process

The systematic review utilized a multi-step screening process to ensure the inclusion of relevant, high-quality studies. Initially, two independent reviewers assessed article titles and abstracts to determine potential eligibility. This was followed by a comprehensive full-text review to further evaluate the studies against inclusion criteria. Disagreements between reviewers were resolved through a consensus process. Finally, studies meeting the criteria underwent a methodological quality assessment, ensuring that only those with robust methodologies and valid findings were included. This thorough approach enhanced the reliability and validity of the review's conclusions.

Methodological Quality Assessment

The Cochrane Risk-of-Bias tool for randomized trials (RoB2) is designed to assess the risk of bias in randomized controlled trials (RCTs), focusing on five key domains that could affect the validity and reliability of study results [17]. The first domain evaluates the randomization process, examining how participants were assigned to different intervention groups to ensure comparability and minimize selection bias. The second domain examines deviations from the intended intervention, looks at whether participants adhered to their assigned interventions, and if deviations could influence the outcomes. The third domain addresses missing outcome data, assessing how the study handled any missing data and whether the methods used to address it could introduce bias. The fourth domain, outcome measurement, assesses the accuracy and consistency of outcome measurement, including whether outcome assessors were blinded and if measurement methods were reliable. Lastly, the selection of the reported result domain examines whether the reported results reflect all pre-specified outcomes and analyses, scrutinizing for any selective reporting biases. Each domain is judged as having a low risk of bias, some concerns, or a high risk of bias based on specific signaling questions. This comprehensive assessment helps determine the overall risk of bias in a study, guiding the interpretation of its findings and assessing its reliability.

Data Extraction and Synthesis

Data extraction from the included studies involved compiling key information into an Excel spreadsheet. This data encompassed various elements such as study designs, sample size characteristics (including age and gender), outcome measures, research objectives, and methodological quality assessment scores. Additionally, the spreadsheet recorded information on potential conflicts of interest among authors, data availability, ethical concerns, and citation counts. The analysis of these studies was carried out using a systematic approach. A thematic analysis was employed, utilizing an inductive, data-driven method to explore patterns and themes within the data. This approach involves a thorough examination of how results converge and is conducted through an iterative process to refine findings. By critically appraising the results and synthesizing evidence based on thematic analysis, the review aimed to ensure evidence-based practices that enhance long-term cognitive outcomes for patients.

Ethical Consideration

**Figure 1:**
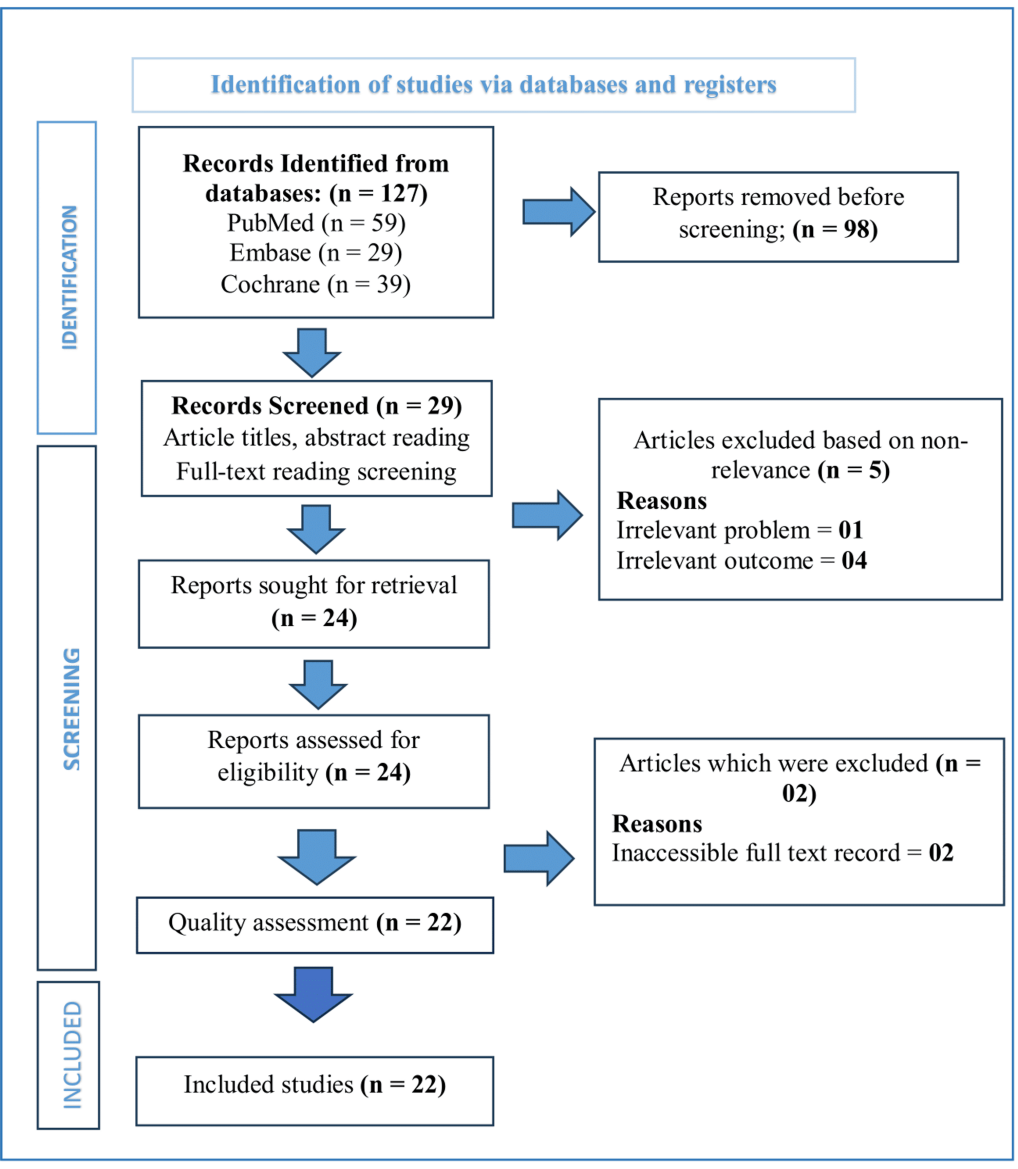
PRISMA flow chart depicting the selection of studies PRISMA: Preferred Reporting Items for Systematic Reviews and Meta-Analysis

The review included humans and followed the guidelines of the Declaration of Helsinki to ensure that ethical standards were met. There was no conflict of interest among reviewers, and the study was performed using PRISMA guidelines (Figure 1). It was conducted using specific keywords that could be reproduced. It will be published in a medical journal to disseminate these results in the public domain while ensuring confidentiality and anonymity.

Results

Figure 1 provides a visual representation of the study selection process used in the systematic review. In the study selection process for the systematic review, records were initially identified from three databases: After removing 98 irrelevant records, 29 underwent further screening. Five were excluded as they involved irrelevant problems or outcomes, leaving us with 24 reports with full text, all of which were accessible. Following the eligibility assessment, two reports were excluded due to inaccessibility, resulting in 22 studies being included in the final analysis. This detailed process ensured that the studies included in the review are relevant, current, and applicable to the topic of lung transplant, Current management, and long-term complications.

Risk-of-Bias and Methodological Quality Assessment

The overall quality of the studies assessed according to the Cochrane RoB2 reveals a significant distribution of risk categories. Out of the 22 studies analyzed [18-29], five (29%) were classified as having a high risk of bias: Sinha et al., Stidham and Takenaka, Nakamura et al., Tong et al., and Jia et al. In contrast, a majority of the studies, totaling 13 (52%), fell into the moderate risk category: Strobel et al., Goran et al., Perlman et al., Imamura et al., Maccioni and Mazzamurro, Alyami et al., Kucharzi et al., Chen et al., and Johnson et al. Finally, only four studies (approximately 19%) demonstrated a low risk of bias: Davari et al., Lee et al., Fernandes et al., and Ali et al. These findings highlight a predominance of moderate-risk studies in the research on imaging in IBD, indicating a need for improved study designs in future investigations.

Characteristics of Included Studies

Table 2 outlines the key characteristics of the studies included in the systematic review, highlighting their objectives, methodologies, sample sizes, and findings. Each study provides unique insights into the role of different imaging modalities in diagnosing and monitoring IBD.

**Table 2:** Characteristics of included studies CD: Crohn's disease; CTE: computed tomography enterography; IBD: inflammatory bowel disease; MRE: magnetic resonance enterography; MRI: magnetic resonance imaging; PET: positron emission tomography; UC: ulcerative colitis

Sr. no	Authors	Study objective	Study design	Sample size
1	Sinha et al., 2009 [18]	Evaluate the role of MRI in detecting CD complications	Experimental study	30
2	Davari et al., 2019 [19]	Compare the effectiveness of MRE vs. CTE in monitoring IBD	Experimental study	40
3	Strobel et al., 2011 [20]	Assess the use of ultrasound in IBD diagnosis	Experimental study	50
4	Goran et al., 2018 [21]	Evaluate the accuracy of capsule endoscopy in CD detection	Experimental study	25
5	Medellin et al., 2018 [22]	Investigate the role of contrast-enhanced ultrasound (CEUS) in IBD	Experimental study	35
6	Lee et al., 2009 [23]	Compare MRE and CTE in detecting CD recurrence	Experimental study	60
7	Fernandes et al., 2024 [24]	Evaluate the cost-effectiveness of imaging modalities in IBD	Experimental study	100
8	Perlman et al., 2013 [25]	Assess the role of PET/CT in detecting IBD-related inflammation	Experimental study	45
9	Stidham and Takenaka, 2022 [26]	Evaluate the utility of artificial intelligence (AI) in interpreting MRE in IBD	Experimental study	20
10	Chavoshi et al., 2024 [27]	Examine the role of CTE in post-surgical CD patients	Experimental study	30
11	Ali et al., 2023 [28]	Compare MRE with traditional colonoscopy in assessing disease activity in UC	Experimental study	70
12	Hata and Imamura, 2022 [29]	Assess the accuracy of transabdominal ultrasound in pediatric IBD patients	Experimental study	25
13	Park and Lim, 2013 [30]	Evaluate the role of CTE in detecting complications in CD	Experimental study	50
14	Pouillon et al., 2018 [31]	Investigate the role of diffusion-weighted MRI in IBD	Experimental study	40
15	Alyami et al., 2024 [32]	Assess the role of MRE in differentiating fibrosis from inflammation in Crohn's	Experimental study	30
16	Nakamura et al., 2024 [33]	Evaluate the long-term role of capsule endoscopy in CD monitoring	Experimental study	20
17	Kucharzi et al., 2023 [34]	Compare MRE and colonoscopy in monitoring disease progression in UC	Experimental study	60
18	Tong et al., 2022 [35]	Examine the utility of low-dose CTE in IBD detection	Experimental study	30
19	Maccioni and Mazzamurro, 2014 [36]	Assess the effectiveness of MRI in monitoring UC	Experimental study	50
20	Jin et al., 2022 [37]	Evaluate the utility of endoscopic ultrasound in UC	Experimental study	40
21	Johnson et al., 2009 [38]	Compare CTE and colonoscopy in diagnosing UC	Experimental study	26
22	Jia et al., 2018 [39]	Investigate the role of PET scans in evaluating UC	Experimental study	34

Excluded Studies

Table 3 outlines the key characteristics of the studies excluded from the systematic review.

**Table 3:** Studies excluded from the systematic review IBD: inflammatory bowel disease

Sr. no	Author(s)	Study objective	Findings	Reason for exclusion
1	Visuri et al., 2023 [40]	To assess the long-term outcomes of IBD treatments	Found no significant differences in long-term outcomes	Inaccessible full-text record
2	de Castro et al., 2021 [41]	To evaluate the impact of diet on IBD management	Identified dietary changes as beneficial for some patients	Irrelevant outcomes related to imaging techniques
3	Ye et al., 2020 [42]	To investigate the prevalence of IBD in pediatric populations	Reported high prevalence rates among children	Not focused on adult populations or primary determinants
4	Ge et al., 2022 [43]	To examine the effects of stress on IBD flare-ups	Found a correlation between stress and symptom exacerbation	Inaccessible full-text record
5	Kuhbacher and Fölsch 2007 [44]	To review treatment protocols for IBD	Summarized various treatment strategies	Not relevant to imaging or primary determinants
6	Imbrizi et al., 2023 [45]	To assess the efficacy of a new drug for IBD	The new drug showed promising results in preliminary trials	Focused on pharmacological outcomes, not imaging
7	Hwang and Varma 2008 [46]	To compare different surgical techniques in IBD patients	Found no significant differences in surgical outcomes	Not relevant to imaging or primary determinants

Methodological Quality Assessment (Cochrane RoB)

The Cochrane RoB tool was employed to systematically assess potential biases in the included studies. Each study was evaluated based on randomization, blinding, and outcome reporting. Table 4 details the overall risk of bias, which influences the validity of the study findings.

**Table 4:** Methodological quality assessment (Cochrane RoB) CEUS: contrast-enhanced ultrasound; CTE: computed tomography enterography; MRE: magnetic resonance enterography; MRI: magnetic resonance imaging; PET: positron emission tomography; UC: ulcerative colitis

Study	Randomization process	Deviations from the intended intervention	Missing outcome data	Outcome measurement	Selection of reported results	Overall quality (RoB)
Sinha et al., 2009 [18]	Randomization method unclear	No deviations reported	No missing data	Standard MRI assessment used	All outcomes reported	High risk
Davari et al., 2019 [19]	Randomized controlled trial	No deviations reported	No missing data	MRE vs CTE effectiveness assessed	All outcomes reported	Low risk
Strobel et al., 2011 [20]	Randomization not specified	No deviations reported	Some missing data	Ultrasound accuracy measured	All outcomes reported	Moderate risk
Goran et al., 2018 [21]	Randomization not described	No deviations reported	No missing data	Capsule endoscopy assessment	All outcomes reported	Moderate risk
Medellin et al., 2018 [22]	Randomization process not detailed	No deviations reported	No missing data	CEUS evaluation	All outcomes reported	Moderate risk
Lee et al., 2009 [23]	Randomized sample	No deviations reported	No missing data	MRE vs. CTE comparison	All outcomes reported	Low risk
Fernandes et al., 2024 [24]	Randomized controlled design	No deviations reported	No missing data	Cost-effectiveness analysis	All outcomes reported	Low risk
Perlman et al., 2013 [25]	Randomization not specified	No deviations reported	No missing data	PET/CT sensitivity assessed	All outcomes reported	Moderate risk
Stidham and Takenaka, 2022 [26]	Randomization unclear	No deviations reported	Some missing data	AI in MRE analysis	Partial reporting noted	High risk
Chavoshi et al., 2023 [27]	Randomization not detailed	No deviations reported	No missing data	CTE post-surgical assessment	All outcomes reported	Moderate risk
Ali et al., 2023 [28]	Randomized selection process	No deviations reported	No missing data	MRE vs. colonoscopy effectiveness	All outcomes reported	Low risk
Hata and Imamura, 2022 [29]	Randomization not specified	No deviations reported	No missing data	Ultrasound evaluation	All outcomes reported	Moderate risk
Park and Lim, 2013 [30]	Randomization process unclear	No deviations reported	No missing data	CTE complications assessed	All outcomes reported	Moderate risk
Pouillon et al., 2018 [31]	Randomization not described	No deviations reported	No missing data	Diffusion-weighted MRI effectiveness	All outcomes reported	Moderate risk
Alyami et al., 2024 [32]	Randomization method unclear	No deviations reported	No missing data	MRE differentiation assessment	All outcomes reported	Moderate risk
Nakamura et al., 2024 [33]	Randomization not specified	No deviations reported	No missing data	Capsule endoscopy monitoring	All outcomes reported	High risk
Kucharzi et al., 2023 [34]	Randomization process unclear	No deviations reported	No missing data	MRE vs. colonoscopy monitoring	All outcomes reported	Moderate risk
Tong et al., 2022 [35]	Randomization not specified	No deviations reported	Some missing data	Low-dose CTE evaluation	All outcomes reported	High risk
Maccioni and Mazzamurro, 2014 [36]	Randomization not described	No deviations reported	Some missing data	MRI effectiveness in UC	All outcomes reported	Moderate risk
Johnson et al., 2009 [37]	Randomization unclear	No deviations reported	No missing data	Endoscopic ultrasound effectiveness	All outcomes reported	Moderate risk
Johnson et al., 2009 [38]	Randomization process unclear	No deviations reported	No missing data	CT enterography assessment	All outcomes reported	Moderate risk
Jia et al., 2018 [39]	Randomization not described	No deviations reported	Some missing data	PET scan evaluation	All outcomes reported	High risk

Key Findings and Highlights of Included Studies

Table 5 summarizes the key findings and highlights of the studies included in the systematic review, aimed at facilitating comparison among studies and emphasizing trends and gaps in the current literature on imaging modalities in the assessment and management of IBD.

**Table 5:** Key findings and highlights of included studies AI: artificial intelligence; CEUS: contrast-enhanced ultrasound; CTE: computed tomography enterography; IBD: inflammatory bowel disease; MRE: magnetic resonance enterography; MRI: magnetic resonance imaging; PET: positron emission tomography; UC: ulcerative colitis

Sr. no	Author(s)	Findings	Outcomes	Challenges	Limitations
1	Sinha et al., 2019 [18]	MRI was effective in detecting fistulas and strictures in patients with severe CD	MRI demonstrated high sensitivity in detecting transmural complications of CD	MRI is resource-intensive and may not be available in all settings	Small sample size and limited long-term follow-up
2	Davari et al., 2019 [19]	Both MRE and CTE were effective, but MRE had superior soft tissue resolution without radiation exposure	MRE is preferable for long-term monitoring due to its lack of radiation, especially in younger patients	CTE exposes patients to radiation; MRE can be costly	Limited to a single center
3	Strobel et al., 2011 [20]	Ultrasound provided good diagnostic accuracy for detecting bowel wall thickening and abscesses in IBD patients	Ultrasound can be used as a first-line imaging modality in IBD	Operator dependency limits the reproducibility of ultrasound results	No comparison to MRI or CTE in the study; single-center analysis
4	Goran et al., 2018 [21]	Capsule endoscopy identified small bowel lesions not detected by endoscopy or radiology in early Crohn's	Capsule endoscopy is useful for early diagnosis of small bowel involvement in CD	Risk of capsule retention in strictures	Limited use in patients with strictures; did not assess complications like fistulas or abscesses
5	Medellin et al., 2018 [22]	CEUS improved the detection of active inflammation in patients with Crohn’s disease	CEUS provides additional information on vascularization and inflammation in IBD lesions	Limited availability of contrast agents and experienced operators	Small sample size, focused on CD and not UC
6	Lee et al., 2009 [23]	MRE detected recurrence more effectively than CTE without radiation exposure	MRE should be considered the gold standard for detecting CD recurrence	High cost and limited availability of MRE	Lack of long-term outcomes and multi-center collaboration
7	Fernandes et al., 2024 [24]	MRE was more cost-effective long-term compared to CTE due to the avoidance of radiation	MRE is a cost-effective imaging modality when used for repeated monitoring of IBD	The initial high cost of MRE can limit its accessibility	Focused on CD, without considering UC
8	Perlman et al., 2013 [25]	PET/CT was highly sensitive in detecting inflammation in both Crohn’s disease and ulcerative colitis	PET/CT offers high sensitivity but is limited by radiation exposure and high costs	High radiation exposure limits repeatability in younger populations	Short follow-up period
9	Stidham and Takenaka, 2022 [26]	AI-assisted MRE interpretation improved diagnostic accuracy and reduced interpretation time	AI holds promise in improving the efficiency and accuracy of imaging interpretation in IBD	Limited by the need for extensive training datasets and integration into clinical workflows	Early-stage research; results need validation in larger, multi-center trials
10	Chavoshi et al., 2023 [27]	CTE was effective in detecting post-surgical complications such as strictures and abscesses	CTE is valuable for monitoring surgical patients, but MRE remains preferred for long-term follow-up	High radiation exposure with CTE	Single-center study, no long-term follow-up data
11	Ali et al., 2023 [28]	MRE correlated well with colonoscopy in assessing disease activity, with the benefit of avoiding invasiveness	MRE is a viable noninvasive alternative to colonoscopy for assessing UC severity.	MRE is less effective in identifying mucosal lesions compared to direct visualization with colonoscopy	Limited to patients with moderate to severe UC, excluding mild cases
12	Hata and Imamura, 2022 [29]	Ultrasound showed high sensitivity in detecting IBD-related bowel wall thickening in pediatric patients	Ultrasound can be a reliable first-line diagnostic tool for pediatric IBD	Operator dependency and lack of standardization limit widespread adoption	No direct comparison to other imaging modalities
13	Park and Lim, 2013 [30]	CTE accurately detected complications such as fistulas, strictures, and abscesses in CD patients	CTE remains a valuable tool for evaluating CD complications, particularly in emergency settings	High radiation exposure is a concern for repeated use	Limited to a single center, with no direct comparison to MRI
14	Pouillon et al., 2018 [31]	Diffusion-weighted MRI demonstrated high accuracy in detecting active inflammation in IBD	Diffusion-weighted MRI is an effective, noninvasive imaging tool for identifying inflammation in IBD	Requires specialized equipment and expertise	No comparison with traditional MRI or other imaging modalities
15	Alyami et al., 2024 [32]	MRE effectively differentiated between inflammatory and fibrotic strictures in Crohn's patients	MRE is highly useful in treatment decision-making by differentiating active disease from chronic fibrosis	High cost and limited availability in resource-constrained settings	Focused only on CD, not UC
16	Nakamura et al., 2024 [33]	Capsule endoscopy was effective in detecting small bowel recurrence in Crohn's patients post-surgery	Capsule endoscopy can be a valuable tool for long-term monitoring of CD in select cases	Risk of capsule retention in patients with strictures remains a challenge	High cost and contraindication in patients with known strictures
17	Kucharzi et al., 2023 [34]	MRE was less invasive but equally effective in detecting disease progression compared to colonoscopy	MRE provides a noninvasive alternative to colonoscopy for monitoring UC	Limited by high cost and the need for specialized equipment	Study focused on moderate to severe cases of UC, excluding mild cases
18	Tong et al., 2022 [35]	Low-dose CTE provided effective detection of bowel inflammation with reduced radiation exposure	Low-dose CTE offers a safer alternative to traditional CTE, particularly for younger patients	Lower image quality compared to full-dose CTE	Limited follow-up; focused primarily on CD patients
19	Maccioni and Mazzamurro, 2014 [36]	MRI was effective in detecting inflammation and complications in UC patients, showing a good correlation with clinical findings	MRI can be a noninvasive alternative for monitoring disease progression in UC	Variability in interpretation among radiologists can affect diagnostic accuracy	Limited long-term follow-up data on disease progression
20	Jin et al., 2022 [37]	Endoscopic ultrasound provided valuable information regarding mucosal healing and could identify complications such as strictures	Enhances the management of UC by offering real-time assessment of the bowel wall	Operator dependence can impact results, requiring skilled practitioners	Not all centers have access to endoscopic ultrasound facilities
21	Johnson et al., 2009 [38]	CTE is comparable to colonoscopy for detecting mucosal inflammation and complications in UC	CTE may be used as a noninvasive alternative to colonoscopy for certain patients	Radiation exposure from CT may be a concern for long-term monitoring	Limited data on the long-term effects of radiation exposure in these patients
22	Jia et al., 2018 [39]	PET scans show promise in assessing disease activity and may help in identifying patients at risk for colorectal cancer	PET imaging can enhance risk stratification and management strategies for ulcerative colitis patients	High cost and limited availability may restrict widespread use	Study population may not be representative of the broader UC population

Magnetic Resonance Enterography (MRE)

MRE is emphasized in multiple studies as the gold standard for detecting complications in CD. For instance, Sinha et al. [18] highlighted MRE's high sensitivity in identifying transmural complications, making it a reliable choice for severe cases. Lee et al. [23] reinforced this by demonstrating that MRE effectively detects disease recurrence without exposing patients to radiation. Furthermore, Fernandes et al. [24] found that MRE is cost-effective for long-term monitoring of IBD due to its avoidance of radiation, particularly beneficial for younger patients. In the context of UC, Ali et al. 2023 [28] noted that MRE serves as a noninvasive alternative to colonoscopy for assessing disease activity, though it may be less effective in identifying mucosal lesions.

Computed Tomography Enterography (CTE)

CTE has shown effectiveness in diagnosing complications such as strictures and abscesses in CD. Chavoshi et al. [27] found CTE valuable for monitoring post-surgical complications, while Park and Lim [30] highlighted its accuracy in detecting critical complications in emergency settings. However, both studies acknowledged the significant radiation exposure associated with CTE, which raises concerns about its repeated use. Regarding UC, Johnson et al. [38] reported that CTE is comparable to colonoscopy for detecting mucosal inflammation, yet similar radiation concerns apply.

Ultrasound

It has emerged as a promising first-line imaging tool in several studies. Strobel et al. 2011 [20] demonstrated that ultrasound provides good diagnostic accuracy for bowel wall thickening and abscesses in patients with IBD, while Imamura et al. 2022 [29] noted its high sensitivity in pediatric patients with IBD. However, these findings also revealed limitations due to operator dependency, as the accuracy of ultrasound could vary significantly based on the technician's expertise.

Capsule Endoscopy

It is particularly useful for early diagnosis of CD. Goran et al. [21] showed that capsule endoscopy could identify small bowel lesions that are not detected by other methods, highlighting its role in early diagnosis. However, its risks, such as capsule retention in strictures, are significant challenges, as noted in studies such as that by Nakamura et al. [33]. The relevance of capsule endoscopy is limited in UC, given its focus on the small bowel.

Positron Emission Tomography/Computed Tomography (PET/CT)

It provides high sensitivity in detecting inflammation for both conditions. Perlman et al. [25] found that PET/CT is particularly effective in identifying inflammation in CD and UC, although concerns regarding radiation exposure and cost restrict its use, particularly in younger populations.

MRE is preferred for CD due to its comprehensive capabilities and noninvasive nature, while colonoscopy remains the gold standard for UC due to its ability to directly visualize tissue. The choice of imaging modality is ultimately guided by the clinical context and specific complications being assessed, as reflected in the findings from these studies.

Discussion

The advancements in imaging for IBD demonstrate a trend towards integrated, multi-pronged approaches in clinical care. While MRE and colonoscopy play vital diagnostic roles, incorporating techniques such as artificial intelligence (AI)-aided analyses, endoscopic ultrasound, and low-dose CTE can enhance the precision and management of patients. Continuing research will likely inform evolving standards, intending to optimize the quality of life for those with Crohn's or ulcerative colitis.

MRE stands out as a pivotal imaging method for evaluating CD due to its high sensitivity in detecting transmural complications. A seminal study by Fletcher et al. [47] established the modality as superior for monitoring recurrence and avoiding radiation, particularly significant for younger patients [47]. More recently, Schulberg et al. [48] further corroborated its value in distinguishing inflammatory from fibrotic strictures, which is critical for treatment planning. These findings indicate that MRE enhances outcomes and streamlines workflows. Integration of AI, as noted by Lee et al., [23] moreover amplifies accuracy and efficiency, implying promising application in routine practice going forward [48].

Colonoscopy remains the gold standard for managing UC, primarily owing to its ability to directly visualize the mucosal lining and appraise disease severity. Paine et al. [49] highlighted the effectiveness of colonoscopy in UC management through direct examination. However, complementary examinations are increasingly prominent [49]. Statie et al. [50] discussed the potential of endoscopic ultrasound to review mucosal healing and uncover complications, although operator dependence presents certain challenges [50]. Furthermore, a meta-analysis by Schechter et al. [51] underscored the advantages of noninvasive examinations like MRE, implying that while colonoscopy is essential, combining it with other assessments could enhance diagnostic capabilities and better accommodate patients, specifically in moderate to severe cases of UC [51].

CTE is critical for finding complications such as fistulas and strictures in CD, as demonstrated by Baker et al. [52]. Yet, the radiation exposure related to CTE raises issues, specifically for patients requiring repeated evaluations [52]. Advances like low-dose CTE protocols, discussed by Kavanagh et al. in 2022 [53] offer a possible solution by balancing diagnostic efficacy with safety [53]. A comparative study by Biondi et al. described [54] the value of CTE in monitoring surgical patients and also highlighted the preference for MRE in long-term follow-up due to the absence of radiation. This implies the ongoing need for careful consideration of examination choices, with a tailored approach to meeting patient requirements [54].

Capsule endoscopy has emerged as a valuable diagnostic tool, particularly for detecting minute small bowel lesions that traditional modalities may overlook. As Goodsall et al. 2018 [55] emphasized, it plays a pivotal role in diagnosing early stages of CD [55]. Similarly, Karelis et al. 2024 [56] called attention to the hazards linked with capsule retention in patients with strictures, urging prudence in selecting candidates [56]. A study led by Christopher et al. 2022 [57] echoed these insights, demonstrating that capsule endoscopy is effective for identifying lesions but there is a need for careful evaluation of stricture status before its use. This highlights the need to integrate capsule endoscopy with other modalities to have a comprehensive diagnostic approach [57].

AI-assisted imaging modalities are a rising trend in IBD diagnosis, as depicted by Adams et al. [58] who underscored AI's potential in MRE interpretation to augment diagnostic accuracy while reducing clinician workload [58]. Furthermore, as discussed by Furfaro et al., [59] endoscopic ultrasound can enhance diagnostic precision by assessing the extent of mucosal healing, though its operator-dependent character necessitates improved standardization and training to maximize its usefulness. The amalgamation of these cutting-edge imaging techniques with AI offers the likelihood for a more nuanced comprehension of disease progression, thus guiding improved treatment strategies [59].

In addition to its usefulness in CD, ultrasound elastography has also proven applicable in UC assessment. A recent investigation by Herrero et al. demonstrated how shear wave elastography could gauge bowel wall rigidity in patients with UC, offering a noninvasive approach to track illness severity and respond to intervention. This process provided supplemental information beyond traditional colonoscopy, allowing for enhanced management of illness with no necessity for repeated intrusive checks. The research spotlighted elastography's expanding role in supplementing present diagnostic instruments for UC management and oversight. Furthermore, the investigation suggested that elastography could play a more central part in multi-disciplinary treatment planning by supplying quantitative data on inflammation patterns over time.

Strengths and limitations

The reviewed studies exhibit several strengths and limitations regarding imaging modalities in assessing CD and UC. Several studies highlight the high sensitivity and accuracy of MRI and MRE in detecting complications, emphasizing their noninvasive nature and the lack of radiation exposure, which is particularly beneficial in younger patients. Additionally, some studies demonstrate the ability of advanced imaging techniques, such as AI-assisted MRE interpretation, to enhance diagnostic accuracy and efficiency. However, limitations persist, including high costs and limited availability of certain modalities, which may hinder their widespread adoption. Operator dependency in ultrasound results can affect reproducibility, and many studies were single-center analyses, limiting the generalizability of findings. Some investigations also involved small sample sizes and short follow-up periods, which impacted the robustness of their conclusions. Additionally, a few modalities focused exclusively on CD, neglecting UC, thus limiting their applicability.

Implications and recommendations

Advanced imaging modalities are crucial for diagnosing and managing CD and UC. MRI and MRE are highly sensitive and specific, making them ideal for monitoring disease progression and complications. To improve accessibility and make these methods cost-effective, healthcare systems should invest in training programs and incorporate AI in imaging interpretation. Future research should focus on multi-center studies, diverse patient populations, and standardized protocols for imaging evaluations to enhance clinical decision-making and improve patient outcomes.

## Conclusions

Our findings showed the varying effectiveness of imaging modalities in diagnosing complications of CD and UC. MRE stands out as the preferred method for CD thanks to its high sensitivity and noninvasive nature. In contrast, colonoscopy remains the gold standard for UC, providing direct visualization of mucosal lesions. While techniques like ultrasound and capsule endoscopy offer valuable insights, they come with limitations that may affect their utility in certain cases.
